# Hyperlipidemia inhibits the protective effect of lisinopril after myocardial infarction via activation of dendritic cells

**DOI:** 10.1111/jcmm.15060

**Published:** 2020-02-19

**Authors:** Yuanji Ma, Leilei Ma, Jiaqi Ma, Runda Wu, Yunzeng Zou, Junbo Ge

**Affiliations:** ^1^ Department of Cardiology Zhongshan Hospital Fudan University Shanghai China; ^2^ Shanghai Institute of Cardiovascular Diseases Shanghai China

**Keywords:** angiotensin‐converting enzyme inhibitor, dendritic cell, hyperlipidemia, myocardial infarction

## Abstract

To investigate the prevention of cardiac remodelling and inflammatory immune response after myocardial infarction (MI) via ACEI regulating dendritic cells (DCs), we explored whether the protective effect of ACEI was repressed under hyperlipidemic environment. In vivo, the survival rate and left ventricular function of the mice were recorded on day 7 after MI. Tissue samples of the myocardium, spleen, bone marrow and peripheral blood were assessed for Ang II concentration, inflammatory cytokines and DCs expression. In vitro, DCs were treated with ox‐LDL + Ang II, simulating the internal environment of MI in ApoE^−/−^ mice to explore the mechanism involved in the DCs maturation and inflammation. Under hyperlipidemic circumstances, we found that the cardioprotective effect of ACEI was attenuated through regulating DCs maturation and inflammation after MI, affecting survival rate and left ventricular function. Effects of lisinopril on the release of spleen‐derived DCs and myocardial infiltration were also reduced under hyperlipidemic conditions. In vitro, immune maturation and inflammation of DCs were further induced by ox‐LDL on the basis of Ang II treatment, as indicated by the upregulation of CD83, CD86, and the expressions of cytokines and chemokines. Furthermore, ox‐LDL could activate TLR4‐MyD88 signalling pathway, promoting IRAK‐4 and NF‐κB. The present study demonstrated that ACEI reduced the recruitment of DCs to the infarct site, leading to a higher survival rate and improved function. However, this effect was inhibited under hyperlipidemic environment. TLR4‐MyD88 signalling pathway may be responsible for the molecular mechanism involved in the immune maturation and inflammation of DCs induced by ox‐LDL.

## 1 INTRODUCTION

Angiotensin‐converting enzyme inhibitor (ACEI) can act directly on myocardial tissue through inhibition of angiotensin II (Ang II) formation, which improves myocardial hypertrophy and fibrosis,^1^ reducing the overall morbidity and mortality of acute myocardial infarction (MI).^2^ However, the protective effects of ACEI may not be beneficial for all patients in clinical practice.^3^

The two most important risk factors associated with the occurrence and development of MI include hyperlipidemia and hypertension. Together, the two can have a strong synergistic effect, while oxidized low‐density lipoprotein (ox‐LDL) and Ang II also play a pivotal role. Cytotoxic ox‐LDL is a risk factor for early ventricular remodelling, affecting cardiac structure and function.^4^, ^5^ Previous studies have shown that the local myocardial tissue concentration of ox‐LDL may be much higher than that of the peripheral circulation, which can directly damage the endothelium and cardiomyocytes, leading to hypertrophic changes.^6^ Other studies have also suggested that serum levels of ox‐LDL antibodies are significantly elevated in patients with MI and are positively associated with MI‐associated mortality.^7^ Similarly, the level of Ang II in the peripheral blood is increased after MI,^8^ which also affects myocardial remodelling in both infarcted and non‐infarcted areas.

Ang II and ox‐LDL have an inseparable relationship.^9^ Ang II and ox‐LDL can increase the expression of ACE in coronary artery endothelial cells. Based on previous research, we have been suggested that ox‐LDL is an important pathological stimulating factor leading to the RAS activation. The aggregation of dendritic cells (DCs) has been observed in arteriosclerotic plaques and infarcted myocardium. Our research team had previously established that ox‐LDL and Ang II^10^ can induce DCs maturation.

Toll‐like receptors (TLRs) are mainly expressed on the surface of monocytes, macrophages and DCs, which can result in the release of inflammatory mediators. TLR4 is the most distinctive member of the TLR family, due to its ability to transduce signals through both the MyD88 signalling pathway and the TRIF signalling pathway during inflammatory response. Previous studies have shown that ox‐LDL induced circulatory and inflammatory response is achieved partially through TLR4,^11^ which suggested its potential role in lipid molecules and inflammation during MI.

Based on the above results, we can envisage that a hyperlipidemic state triggers immune maturation and migration of DCs, thereby affecting the myocardial protective effects of ACEI.

## 2 MATERIALS AND METHODS

### 2.1 Animals

C57BL/6 and ApoE^−/−^ mice, with an average age of 8‐10 weeks, fed on a control diet and high‐cholesterol diet, respectively, were obtained from the Animal Administration Center of Fudan University. Mice were treated with lisinopril at a dose of 100 mg/L^12^ via drinking water, which was initiated 2 days before MI and continued for 7 days thereafter.

Myocardial infarction was induced by permanent coronary ligation at 8‐10 weeks of age. All procedures and protocols were approved by the Institutional Review Board of Zhongshan Hospital, Fudan University and Shanghai Institutes for Biological Sciences‐CAS (A5894‐01) and were conducted in conformity with the Public Health Service Policy on Humane Care and Use of Laboratory Animals. Splenectomy was performed at the time of MI as well.

### 2.2 Cell culture and treatments

Bone marrow‐derived dendritic cells (BMDCs) obtained from C57BL/6 mice (about 6‐8 weeks) were cultured in RPMI 1640 media supplemented with 10 ng/mL granulocyte‐macrophage colony‐stimulating factor (GM‐CSF) and 1 ng/mL IL‐4 at 37°C in 5% humidified CO_2_ for 4 hours. Non‐adherent cells were replaced with fresh medium every 2 days. On culture day 7, the supernatant of necrotic cells from cardiomyocytes (HL‐1) was added to DCs suspensions for 24 hours, then treated with Ang II (100 nmol/L^10^, ^13^; Sigma‐Aldrich) alone or with additional ox‐LDL (50 μg/mL^14^; Sigma‐Aldrich). Necrotic cells were used as control. In the inhibitor experiment, the cells were exposed to several inhibitors for 1 hour.

### 2.3 Myocardial infarction protocol

Mice were anesthetized by inhalation of isoflurane and intubated with a 22‐G intravenous catheter, followed by full anesthetization with 1.0%‐2.0% isoflurane gas and mechanical ventilation with a positive pressure ventilator. The heart was exposed through a left thoracotomy, and MI was induced by ligating the left coronary artery with an 8‐0 nylon suture. Successful ligation was confirmed when the anterior wall of the left ventricle turned pale. Mice that died within 24 hours after the operation were excluded from further analysis. Sham‐operated animals underwent the same procedure without ligation of the coronary artery.

### 2.4 Preparation of cardiac, splenic and peripheral blood cells

Mice were killed on day 7 after MI (n = 5‐6 mice per group). Spleen was removed, triturated in HBSS (Mediatech, Inc) at 4°C with the end of a 3 mL syringe and filtered through a 100 μm nylon mesh (BD Biosciences). The cell suspension was centrifuged at 300 g for 10 minutes at 4°C. Heart tissue was harvested, minced and digested by collagenase II (Sigma) at a concentration of 0.5 mg/mL in 37°C for 30 minutes. Single‐cell suspension was screened with 40 μm cell strainers (BD Biosciences). The cells were washed and resuspended by HBSS containing 2% BSA. Total spleen and cardiac cell numbers were determined with Trypan blue (Mediatech, Inc). Peripheral blood was drawn via cardiac puncture and subjected to red cell lysis with ACK lysing buffer (150 mmol/L NH_4_Cl, 10 mmol/L KHCO_3_, 10 mmol/L EDTA) and three washes with PBS buffer (PBS containing 1% FCS and 5 mmol/L EDTA).

### 2.5 Flow cytometry

A subset of six mice per group was used for flow cytometry analysis. Cell suspensions were incubated with a mixture of antibodies (anti‐CD11c‐PE, anti‐CD45‐FITC, anti‐CD83‐PE, anti‐CD86‐FITC; BD Biosciences) at 4°C for flow cytometry analysis to determine the percentage of DCs in hearts, spleens and peripheral blood, respectively. Samples were loaded after two washes with PBS (2% BSA), and the raw data were analysed performed with a flow cytometer (Beckman).

### 2.6 Western blotting

Protein samples were fractionated with 12% SDS‐PAGE (Invitrogen, USA), then transferred into polyvinylidene fluoride membranes (Millipore). The membranes with blotted protein were blocked, followed by probing with TLR4, MyD88, TRIF, NF‐κB, phospho‐NF‐κB, IκB, phospho‐IκB (Cell signal technology), IRAK‐4, phospho‐IRAK‐4 (Santa Cruz) antibodies at 4°C overnight. The membranes were washed and incubated with horseradish peroxidase‐conjugated secondary antibody. Immunoreactive proteins were identified using Super Signal West Pico Chemiluminescence Substrate (Thermo). Densitometric analysis of Western blots was performed with the use of Image J software. GAPDH was used as loading control.

### 2.7 Quantitative PCR

The spleen was examined for expression of markers of inflammatory cytokines, tumour necrosis factor‐α (TNF‐α), interleukin (IL‐6), IL‐10 and chemokine (C‐C motif) receptor 7 (CCR7).

### 2.8 Enzyme‐Linked Immunosorbent Assay

The supernatant of the cultured BMDCs was harvested and stored at −70°C. The cytokine concentrations of TNF‐α and IL‐6 were analysed using enzyme‐linked immunosorbent assay (ELISA) kits (R&D Systems) according to the manufacturer's instructions.

Ang II concentration of the blood was determined with Ang II ELISA (Cayman Chemical) according to the manufacturer's instructions.

### 2.9 Statistical analyses

All statistical analyses were performed with SPSS 22. Continuous data were presented as mean ± SD. Statistical comparisons between two groups were evaluated by Student's *t* test and corrected by ANOVA for multiple comparisons. Survival rates were compared by the Kaplan‐Meier method and analysed with the log‐rank test. A *P‐*value of <.05 was considered statistically significant.

## 3 RESULTS

### 3.1 Comparison of lipid profiles 7 days after myocardial infarction

**Figure 1:**
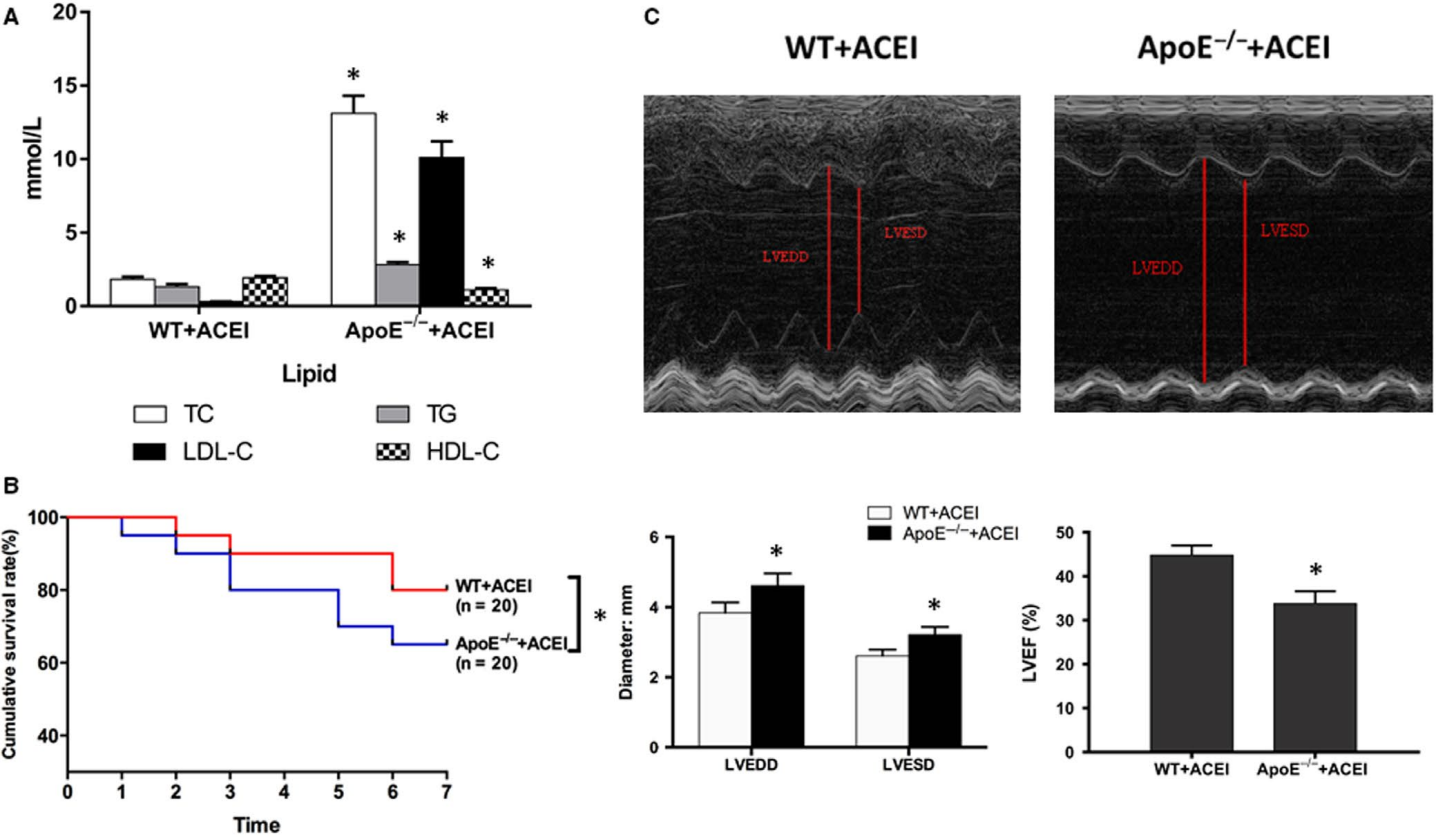
Cardiac function measured by echocardiography on day 7 post‐surgery. A, Comparison of lipid profiles 7 days after myocardial infarction. B, Kaplan‐Meier survival analysis. Percentage of surviving mice after MI was plotted, comparison between‐group difference was tested by the log‐rank test. C, Representative M‐mode images from individual groups. Measurements shown are LVEDD, LVESD and LVEF in the different treatment groups. The data are shown as the mean ± (SD) (n = 6). *P < .05 vs WT ACEI + MI

Total cholesterol (TC), triglyceride (TG), low‐density lipoprotein cholesterol (LDL‐C) and high‐density lipoprotein cholesterol (HDL‐C) were detected on day 7 post‐MI. The results showed that the blood lipid levels of ApoE^−/−^ mice were significantly higher than those of WT mice, including TC, LDL‐C and TG, while HDL‐C level was significantly lower (Figure 1A, *P* < .05). Variables such as presence of MI, treatment with lisinopril and spleen resection did not affect the blood lipid levels of C57BL/6J mice.

### 3.2 Effect of hyperlipidemia on survival after myocardial infarction

The survival rate on day 7 post‐MI was significantly higher in the lisinopril and spleen resection group, compared to control (80% or 75% vs 52.4%, *P* < .05). The survival rate of the sham operation group was 100%. In comparison with the WT group, hyperlipidemia significantly reduced post‐MI survival rate and inhibited the protective effect of lisinopril on mortality (Figure 1B, 65% vs 80%, *P* < .05).

### 3.3 The effect of hyperlipidemia on the protective effect of lisinopril

On day 7 post‐MI, the cardiac function and ventricular lumen diameter of the mice were assessed via echocardiography. Compared with the sham operation group, left ventricular systolic end‐diastolic diameter (LVESD) and left ventricular diastolic end‐diastolic diameter (LVEDD) were significantly increased on day 7 after MI, while left ventricular ejection fraction (LVEF) was significantly decreased (*P* < .05). Intervention with lisinopril and spleen resection significantly reduced LVESD and LVEDD, which improved LVEF and cardiac function (*P* < .05). Given the same dose of lisinopril, LVESD and LVEDD were significantly increased in ApoE^‐/‐^ mice after MI compared with WT mouse, while LVEF was significantly weakened (Figure 1C, *P* < .05). This indicated that hyperlipidemia detriments the protective effect of lisinopril after MI.

### 3.4 Effects of lisinopril on the release of spleen‐derived dendritic cells and myocardial infiltration under hyperlipidemic conditions

We wanted to explore to what extent the impact on the DCs flux contributes to the overall benefits of lisinopril. We thus neutralized the splenic DCs by splenectomy at the time of coronary ligation. As in lisinopril treated mice, this results in a reduced availability of splenic DCs, but leaves non‐spleen targets of lisinopril untouched. Hence, lisinopril can reduce the infiltration of CD11c^+^CD45^+^DCs into myocardial tissue by inhibiting the release of spleen‐derived DCs after MI. However, under hyperlipidemic conditions, a significant decrease in spleen DCs and increase in peripheral blood DCs were observed in ApoE^−/−^ mice, compared to WT mice (*P* < .05). In ApoE^−/−^ mice, ACEI also had a beneficial effect on DCs recruitment, albeit to a lesser degree than treatment with splenectomy. This indicated that the inhibitory effect of lisinopril on the release of DCs from the spleen was weakened under hyperlipidemic state.

**Figure 2:**
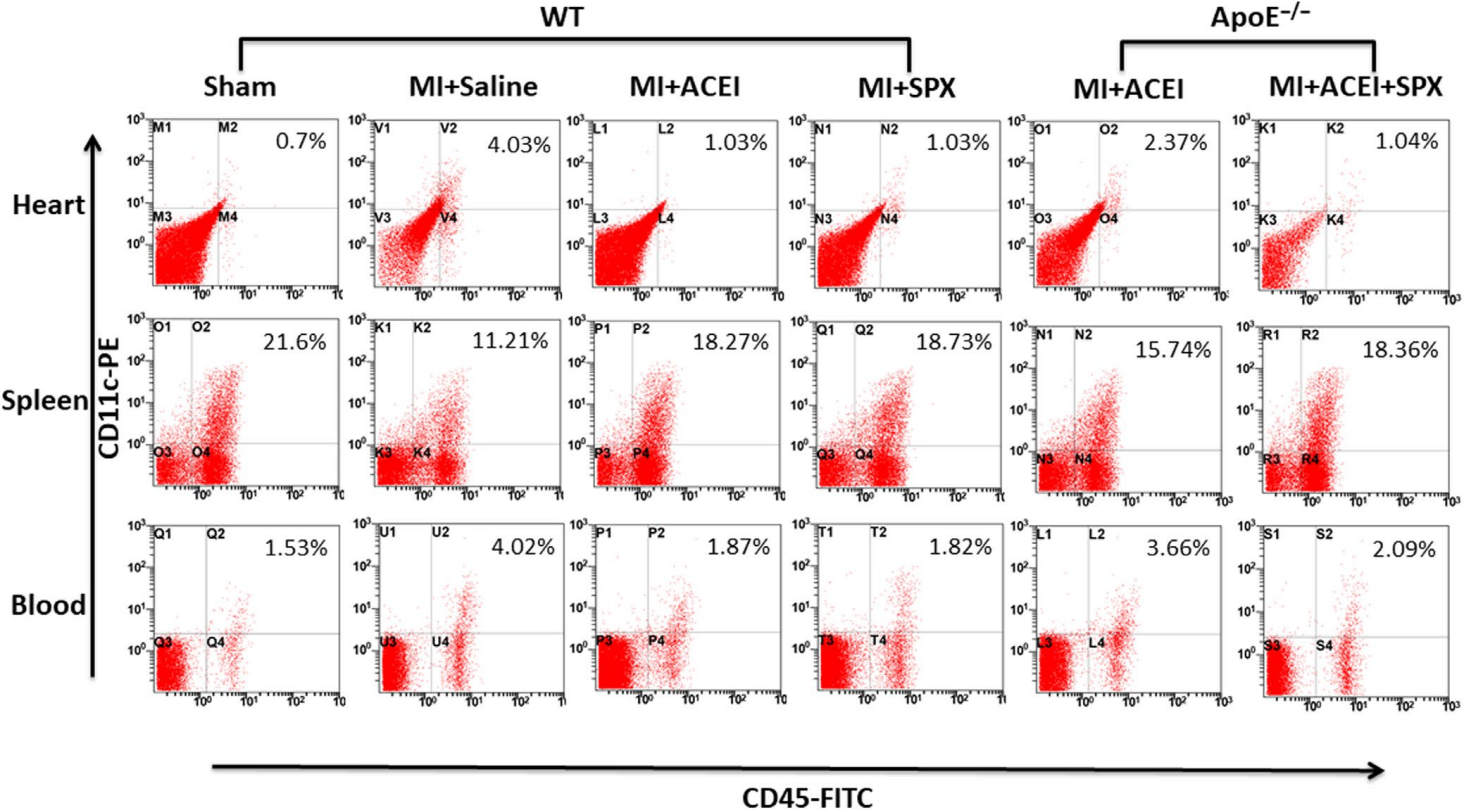
Hyperlipidemic status affects the inhibition of DCs infiltration in myocardium after MI by Lisinopril. Representative dot plots of CD45‐FITC/CD11c‐PE profile of mice 7 days after MI. Numbers on the plots indicated the percentage of CD45^+^CD11c^+^ DCs of total living cells in the heart, spleen and blood. MI and SPX stand for myocardial infarction and splenectomy, respectively

To further demonstrate whether spleen‐derived DCs infiltrated the infarcted myocardium under hyperlipidemic conditions, flow cytometry was used to detect the expression levels of CD11c^+^CD45^+^DCs in myocardial tissues. The results showed that the number of CD11c^+^CD45^+^DCs in the myocardium of ApoE^‐/‐^ group was significantly increased compared with WT group, and the results were statistically significant (Figures 2 and 4A, *P* < .05).

### 3.5 Effects of lisinopril on maturation and inflammatory cytokines of spleen‐derived DCs under hyperlipidemic conditions

Infiltration of DCs plays an important role in the development of ventricular remodelling after MI. Spleen‐derived DCs in the MI group significantly up‐regulated the expression of mature marker CD83 and costimulatory molecule CD86. Meanwhile, qPCR results showed an increase in inflammatory cytokines TNF‐α and IL‐6, indicating that the myocardial infarction can significantly enhance immune maturation of DCs, augment antigen presentation, and participate in inflammatory response of post‐infarction ventricular remodelling, which further hinders the recovery of cardiac function after MI. Lisinopril can significantly inhibit CD83, CD86, TNF‐α, IL‐6, and CCR7 expression and upregulate IL‐10 expression.

**Figure 3:**
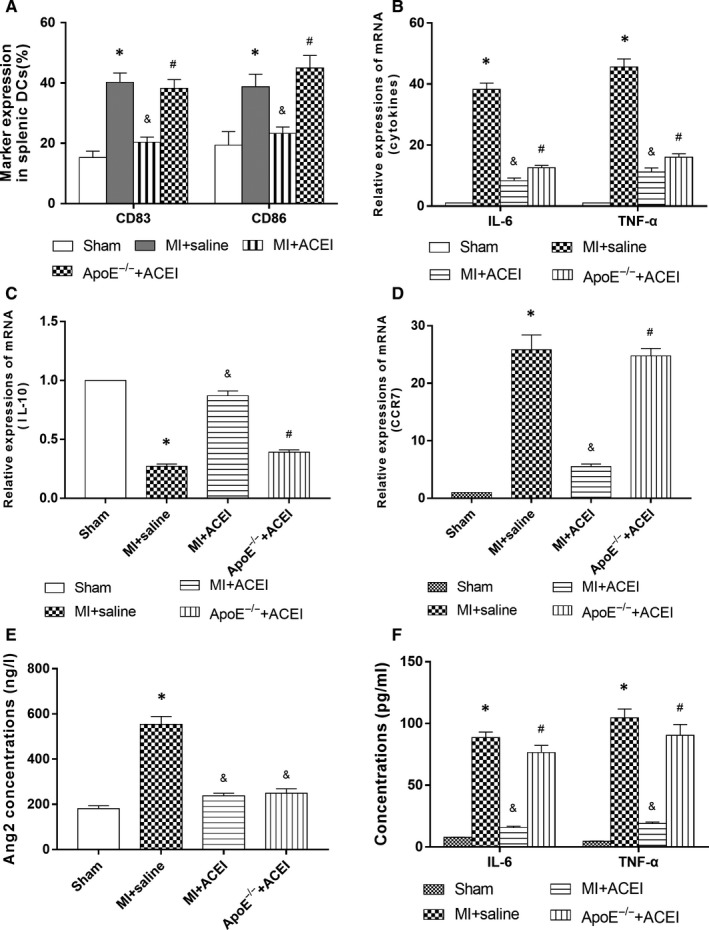
A, Effect of hyperlipidemia on inhibition of splenic DCs maturation by lisinopril in mice. CD80, CD86 and CD83 expression as maturation marker was assessed by flow cytometry. B, C, D, qPCR detection of hyperlipidemia affecting the expression of DCs cytokines and chemokines in spleen of mice. E, F, Expression of secretion of Ang II and inflammatory cytokines in the peripheral circulation under hyperlipidemic conditions by ELISA. *P < .05 vs Sham; &*P* < .05 vs Saline MI; #*P* < .05 vs WT ACEI + MI

The expression of CD83 and CD86 in ApoE^−/−^ mice was up‐regulated under hyperlipidemic conditions. qPCR results also revealed an increase of TNF‐α, IL‐6 and CCR7, but an decrease of IL‐10 in ApoE^‐/‐^ mice, indicating that hyperlipidemia affected the inhibitory effects of lisinopril on the maturation of spleen‐derived DCs and inflammatory response after MI (Figure 3A‐D).

### 3.6 Effects of lisinopril on the secretion of Ang II and inflammatory cytokines in the peripheral circulation under hyperlipidemic conditions

In order to establish that lisinopril inhibited the release of spleen‐derived DCs by decreasing Ang II levels and is not associated with Ang II, the level of Ang II in peripheral circulation after MI was measured. The concentration of Ang II in the peripheral circulation of the MI group was significantly higher than that of the sham‐operated group, while intervention with lisinopril significantly reduced Ang II levels (*P* < .05). Under hyperlipidemic state, the concentration of Ang II in the peripheral circulation was not affected, in comparison to WT mice (Figure 3E, *P* > .05), indicating that the inhibitory effect of lisinopril on the release of spleen DCs under hyperlipidemic conditions was not dependent on the level of Ang II, but rather via other pathways.

Based on previous results, lisinopril can inhibit the release of spleen DCs after MI under hyperlipidemic conditions, which reduced DCs infiltration in the infarcted myocardium, inhibited immune maturation of spleen DCs and down‐regulated expression of inflammatory cytokines. The effect of lisinopril on the secretion of inflammatory cytokines after MI was further tested. The results showed that given the same dose of lisinopril, the concentration of TNF‐α and IL‐6 in high‐fat ApoE^−/−^ mice was significantly higher than that of the WT mice (Figure 3F, *P* < .05), suggesting that a high‐fat state attenuated the inhibitory effects of lisinopril on the secretion of inflammatory cytokines. Collectively, hyperlipidemia combined with MI can result in a systemic inflammatory cascade, which is an important mechanism in promoting ventricular remodelling after infarction. Fortunately, lisinopril can effectively inhibit inflammatory response after myocardial infarction.

### 3.7 ox‐LDL further enhanced DCs migration, maturation and secretion of inflammatory cytokines

**Figure 4:**
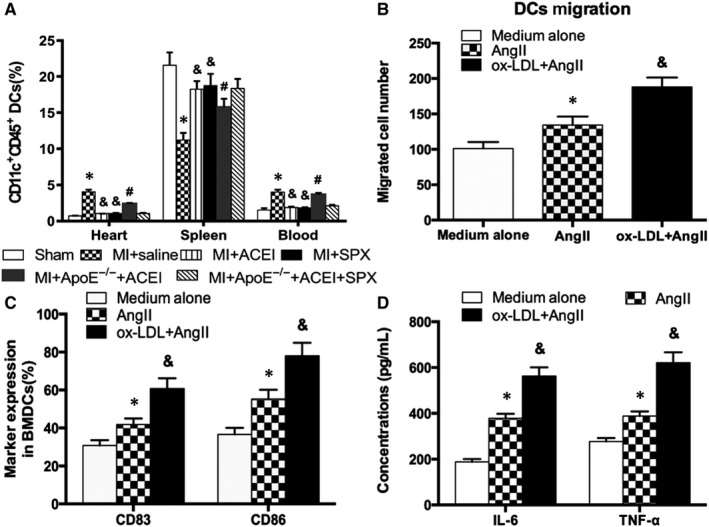
A, Quantitation of CD45^+^CD11c^+^ DCs by flow cytometry in the heart, spleen and blood after MI. The data are shown as mean ± (SD) (n = 5‐6); *P < .05 vs Sham; &*P* < .05 vs Saline MI; #*P* < .05 vs WT ACEI + MI; B, ox‐LDL enhances the migration of DCs on the basis of Ang II. C, D, ox‐LDL further induces DCs maturation and secretion of inflammatory cytokines. *P < .05 vs medium alone; &*P* < .05 vs Ang II

The migration ability of DCs is dependent on the infiltration of infarcted myocardium. However, under hyperlipidemic conditions, this effect is often attenuated. Ox‐LDL and Ang II were used to mimic the internal environment of RAS activation after MI under hyperlipidemic conditions. The scratch injury model was used to study cell migration. Given the same supernatant of necrotic cardiomyocytes, DCs were treated with Ang II, ox‐LDL + Ang II after the scratch test, then photographed after 24 hours and counted for cell migration. The results showed that Ang II could enhance migration of DCs, while ox‐LDL could further induce migration of DCs on the basis of Ang II (Figure 4B, *P* < .05). Results also showed that Ang II promotes high expression of CD83 and CD86 in DCs. Similarly, ox‐LDL could further induce the expression of CD83 and CD86 on the basis of Ang II (Figure 4C, *P* < .05), indicating that a high‐fat internal environment can further stimulate the inflammatory mechanism in vivo and aggravate myocardial inflammatory response after infarction. The expression of inflammatory cytokines TNF‐α and IL‐6 in cell supernatant was detected by ELISA. The results showed that ox‐LDL could further promote the secretion of TNF‐α and IL‐6 on the basis of Ang II (Figure 4D, *P* < .05). A hyperlipidemic state can promote the release of inflammatory cytokines of DCs and aggravate inflammatory response.

### 3.8 ox‐LDL + Ang II can induce DCs activation of TLR4/MyD88‐NF‐κB signalling pathway

**Figure 5:**
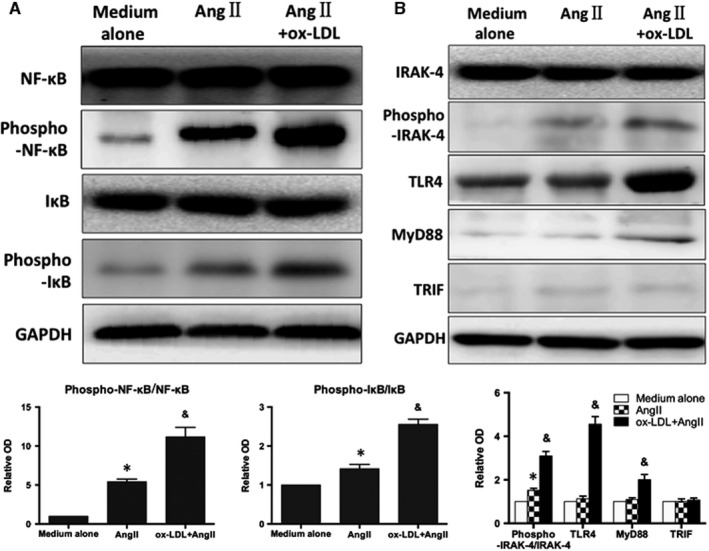
ox‐LDL + Ang II can induce DCs activation of TLR4/MyD88‐NF‐κB signalling pathway. A, Representative immunoblots and the results of quantitative analysis of IκB and NF‐κB phosphorylation in the infarcted heart. B, Western blot shows the expression of TLR4, IRAK‐4, phospho‐IRAK‐4, MyD88 and TRIF after intervention with Ang II alone and ox‐LDL + Ang II. Data were represented as mean ± SD (n = 3). *P < .05

NF‐κB is the main signalling pathway responsible for mediating inflammation. The results showed that ox‐LDL + Ang II can further induce IκB and NF‐κB phosphorylation (Figure 5A, *P* < .05) compared with administration of Ang II alone, which promoted inflammation by stimulating nuclear transcription.

DCs surface TLR4 plays a central role in promoting immunity and is closely related to the activation of NF‐κB signalling pathway and immune maturation of DCs. Therefore, the expression of TLR4, IRAK‐4, phospho‐IRAK‐4, MyD88 and TRIF was examined after intervention with Ang II alone and ox‐LDL + Ang II. Ox‐LDL up‐regulated the expression of TLR4 and MyD88 on the basis of Ang II, as well as phosphorylation of IRAK‐4 (downstream of TLR4) (Figure 5B, *P* < .05). However, little effect on TRIF expression was observed (*P* > .05). This indicated that ox‐LDL further activated the TLR4‐MyD88‐IRAK‐4 signalling pathway on the basis of Ang II, but does not affect the TRIF signalling pathway.

**Figure 6:**
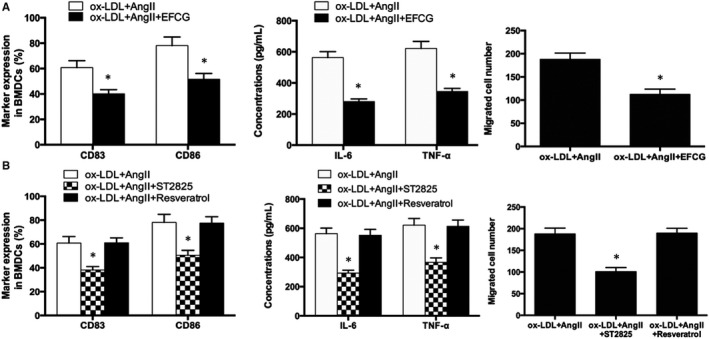
A, TLR4 inhibitor down‐regulates ox‐LDL + Ang II‐induced migration, maturation and secretion of inflammatory factors of DCs, and B, MyD88 inhibitor. *P < .05

To further demonstrate the mechanism by which TLR4/MyD88‐NF‐κB pathway is involved in immune maturation and inflammatory responses of DCs, we intervened ox‐LDL + Ang II‐induced DCs with TLR4 inhibitor EFCG, MyD88 inhibitor ST2825 and TRIF inhibitor Resveratrol. EFCG and ST2825 could down‐regulate the expression of CD83 and CD86, respectively (*P* < .05), and inhibit the secretion of inflammatory cytokines and DCs migration (*P* < .05). However, TRIF inhibitor Resveratrol showed no differences before and after intervention, further validating that TLR4 mediates immune maturation and inflammatory response of DCs through the MyD88 pathway (Figure 6A,B).

**Figure 7:**
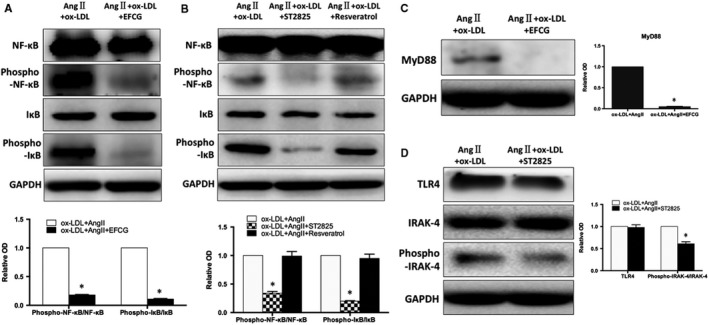
A, TLR4 inhibitor down‐regulates the activation of NF‐κB induced by ox‐LDL + Ang II. B, Changes of NF‐κB phosphorylation after inhibition of MyD88 and TRIF, respectively. C, D, The relationship between TLR4 and MyD88. *P < .05

Simultaneously, the inhibition of TLR4 and MyD88 significantly decreased the phosphorylation of NF‐κB and IκB, and the NF‐κB signalling pathway was also inhibited (*P* < .05). The degree of phosphorylation of NF‐κB and IκB was not affected by TRIF inhibitor Resveratrol. This indicated that TLR4 is achieved via the MyD88 pathway rather than the TRIF pathway during activation of NF‐κB pathway via DCs after treatment with ox‐LDL + Ang II (Figure 7A,B, *P* < .05). TLR4/MyD88‐NF‐κB pathway is involved in the process of DCs maturation and inflammatory response in the development and progression of myocardial infarction with concurrent hyperlipidemia.

### 3.9 Relationship between TLR4 and MyD88

TLR4 inhibitor EFCG was used to interfere with ox‐LDL + Ang II‐induced DCs, which resulted in inhibition of MyD88 expression (Figure 7C, *P* < .05). Conversely, when switched to MyD88 inhibitor ST2825, phosphorylation of IRAK‐4 was reduced (*P* < .05), while TLR4 expression showed a downward trend, but the difference was not statistically significant (Figure 7D, *P* > .05). This indicated that MyD88 is located downstream of TLR4 and is regulated by TLR4. The phosphorylation of IRAK‐4 is regulated by TLR4 regulates via the MyD88 pathway, thereby promoting NF‐κB nuclearization and transcription of inflammatory cytokines.

## 4 DISCUSSION

Inflammatory response is an important part of ventricular remodelling after myocardial infarction.^15^ As a full‐time antigen‐presenting cell and initiator of immune‐inflammatory response in vivo,^16^ DCs play an important role in the myocardial inflammation. Studies from Science^17^ have demonstrated that monocytes and DCs clusters in the spleen infiltrate the infarcted myocardium and participate in post‐infarction ventricular remodelling. Naito K et al^18^ reported that the accumulation of mature DCs in the infarcted myocardium was associated with ventricular remodelling and deterioration of cardiac function after MI.

Abnormal blood lipid metabolism is closely related to the morbidity and mortality of many diseases. Dyslipidemia can directly lead to development of certain diseases and can indirectly affect other disease through immune regulation.^19^ Previous studies have confirmed that high‐fat state can activate DCs, promote inflammatory reactions, and alter the migration of DCs.^20^ After DCs activation, they are converted into foam cells by phagocytosis of ox‐LDL, promoting early atherosclerosis. While, late‐stage DCs are immersed in the atherosclerotic plaque from the outer membrane, which contributes to the rupture of the coronary plaque, leading to acute MI.

In this study, we used a high‐fat diet ApoE^−/−^ mice for coronary artery ligation, so MI is induced in a chronic low‐inflammatory environment, which can be used to simulate the internal environment of AMI patients. The mechanism of coronary atherosclerotic plaque rupture is often due to hyperlipidemia.

In the present study, we found that treatment with lisinopril as well as splenectomy reversed the number of recruited DCs and the quantity of infarcted tissue to levels seen in WT mice, which demonstrated that chronic inflammation after MI promotes immune maturation and inflammatory response of DCs, as well as release of DCs from the spleen and infiltration into infarcted myocardium. While, lisinopril can impede DCs maturation and inflammatory response, inhibit release of DCs from the spleen into the infarcted myocardium, thereby improving inflammatory response after MI. However, in a high‐fat state, this effect of lisinopril was attenuated, indicating that hyperlipidemia can further activate immune maturation and inflammatory expression of DCs. This phenomenon can partially antagonize the inhibitory effect of lisinopril on spleen DCs, thereby aggravating inflammation after MI. Meanwhile, the myocardial protective effect of lisinopril on MI mice was also attenuated under hyperlipidemic conditions, including a decline in cardiac ejection fraction and increase in mortality. The levels of Ang II in peripheral blood of ApoE^−/−^ mice and C57 mice were comparable, indicating that the inhibitory effect of hyperlipidemia on ACEI was not mediated by Ang II.

Among the many risk factors affecting the incidence and development of AMI, hyperlipidemia and hypertension are undoubtedly the two most important risk factors and together have a strong synergistic effect. ox‐LDL and Ang II also play a crucial role in disease development. Cytotoxic ox‐LDL is a risk factor for early ventricular remodelling, affecting cardiac structure and function.^4^ To further reveal the immune maturation and inflammatory response of DCs under hyperlipidemic conditions and the specific mechanism by which lisinopril is attenuated by DCs, Ang II and ox‐LDL were used to intervene with DCs in hyperlipidemic MI mice, which closely simulated the internal environment of RAS activation. The immune maturation and inflammatory response were observed, in which ox‐LDL further induced DCs migration, immune maturation and secretion of inflammatory cytokines on the basis of Ang II. The results were consistent with previous animal experiments. Previous studies have revealed^21^ the family of TLRs on the surface of DCs can recognize PAMP, initiate early responses to human pathogens, which leads to the release of inflammatory mediators. Among them, TLR4 is the most distinctive can signal immune response through the MyD88 signal or the TRIF signalling pathway. It can also recognize exogenous ligands and endogenous ligands, as well as activate NF‐κB signalling pathway and is most closely related to the immunological maturation of DCs. NF‐κB not only mediates downstream signalling of TLR4, but also up‐regulates TLR4 expression in reverse. There may be a positive feedback process between the two.^22^

In vitro, the phosphorylation of NF‐κB/IκB was significantly increased by simultaneous intervention of Ang II and ox‐LDL, suggesting that NF‐κB pathway is involved in DCs immune maturation and inflammatory response induced by ox‐LDL. ox‐LDL + Ang II can promote activation of TLR4/MyD88 and phosphorylation of IRAK‐4 in DCs, while TRIF expression was not enhanced when treated with ox‐LDL + Ang II. However, a significant downregulation of immune‐synthesis and inflammatory response of DCs was observed with ox‐LDL + Ang II, as well as inhibition NF‐κB and IκB phosphorylation, suggesting that TLR4 induces DCs maturation. TLR4 plays an important role in the secretion of inflammatory cytokines. MyD88 inhibitor ST2825 was used for DCs intervention, and the phosphorylation of NF‐κB and IκB was down‐regulated. Meanwhile, TRIF inhibitor Resveratrol did not affect phosphorylation of NF‐κB and IκB. In addition, the relationship between TLR4 and MyD88 was investigated. TLR4 inhibitor EFCG was used to interfere with ox‐LDL + Ang II‐induced DCs. The results suggest that the expression of MyD88 was inhibited. On the contrary, MyD88 inhibitor ST2825 decreased the expression of TLR4 is, but the difference was not statistically significant. MyD88 is located downstream of TLR4 and is regulated by TLR4. The expression of TRIF was not significantly different under the action of two inhibitors. These results indicate that TLR4 is achieved by the MyD88 pathway rather than the TRIF pathway during activation of the NF‐κB pathway via DCs induced by ox‐LDL + Ang II. Therefore, we conclude that TLR4‐MyD88‐NF‐κB pathway is involved in the process of DCs immune maturation and inflammatory response.

## CONFLICT OF INTEREST

The authors declare no competing financial interests.

## DISCLOSURE

The abstract of this manuscript has been presented at 2019 ESC Congress, Paris, France, August 31‐September 4.

## Data Availability

The data that support the findings of this study are available from the corresponding author upon reasonable request.
